# Metformin suppresses pro-inflammatory cytokines in vitreous of diabetes patients and human retinal vascular endothelium

**DOI:** 10.1371/journal.pone.0268451

**Published:** 2022-07-08

**Authors:** Yue Li, Shawn Gappy, Xiuli Liu, Therese Sassalos, Tongrong Zhou, Andrew Hsu, Alice Zhang, Paul A. Edwards, Hua Gao, Xiaoxi Qiao

**Affiliations:** Department of Ophthalmology, Henry Ford Hospital, Detroit, Michigan, United States of America; University of Utah, UNITED STATES

## Abstract

Metformin is a traditional anti-hyperglycemic medication that has recently been shown to benefit vascular complications of diabetes via an anti-inflammatory mechanism other than glycemic control. This study aims to test the hypothesis that metformin suppresses diabetic retinopathy (DR) associated intraocular inflammation. Human vitreous from control and proliferative diabetic retinopathy (PDR) patients with or without long-term metformin treatment (> 5 years) were collected for multiple inflammatory cytokines measurements with a cytokine array kit. The vast majority of the measurable cytokines in PDR vitreous has a lower level in metformin group than non-metformin group. Although the p values are not significant due to a relatively small sample size and large deviations, the 95% confidence interval (CI) for the mean difference between the two groups shows some difference in the true values should not be neglected. Using quantitative ELISA, soluble intercellular adhesion molecule -1 (ICAM-1) and monocyte chemoattractant protein -1 (MCP-1) presented with significantly lower concentrations in metformin group versus non-metformin group. Metformin group also has significantly less up-regulated cytokines and diminished positive correlations among the cytokines when compared to non-metformin group. Possible role of AMP-activated protein kinase (AMPK) and nuclear factor kappa-light-chain-enhancer of activated B cells (NF-κB) in metformin’s anti-inflammatory effects were studied in human retinal vascular endothelial cells (hRVECs) cultured in normal glucose (NG) and high glucose (HG) conditions. Metformin inhibited HG-induced ICAM-1, IL-8, and MCP-1 via AMPK activation, whereas pharmacological AMPK inhibition had no effect on its inhibition of NF-κB p65, sICAM-1, and tumor necrosis factor-α (TNF-α). Metformin-induced suppression of the inflammatory cytokines could also be mediated through its direct inhibition of NF-κB, independent of AMPK pathway. This is a proof-of-concept study that found metformin treatment was associated with reduced inflammatory responses in vitreous of diabetes patients and retinal vascular endothelial cells, supporting the rationale for using metformin to treat DR at an early stage.

## Introduction

Diabetic retinopathy (DR) is the leading cause of vision loss among working age population in the United States [1]. Diabetes management has traditionally relied on glycemic control, which does not adequately prevent or delay the advancement of DR [2]. When non-proliferative diabetic retinopathy (NPDR) advances to proliferative diabetic retinopathy (PDR), laser photocoagulation or intravitreal injection of VEGF antagonists will be applied. Despite pan-retinal photocoagulation and anti-VEGF therapy, patients with PDR experience a variety of visual complications, including severe vision loss. There is a pressing need to find or develop treatments that can improve control of the early pathologies of DR and avoid vision loss in DR patients.

Numerous clinical and experimental research have suggested that chronic inflammation plays a critical role in the early stages of DR, when capillary damage, retinal ischemia, and vascular hyperpermeability occur [3, 4]. Inflammatory cytokines such as interleukins (ILs), tumor necrosis factor (TNF-α), monocyte chemoattractant protein-1 (MCP-1), and intercellular adhesion molecule-1 (ICAM-1) [5] were detected at significantly increased levels in serum and ocular tissue from DR patients, and were found to be associated with early pathognomonic features of DR [3, 6–8]. They could be used as targets for early DR treatment.

Since the 1950s, metformin has been used as the first-line treatment for diabetes. Along with glycemic control, long-term metformin use has been shown to protect against diabetic angiopathy, including DR, in clinical trials such as the United Kingdom Prospective Diabetes Study (UKPDS) [9] and Diabetes Prevention Program Outcomes Study (DPPOS) [10], which was not observed with other diabetes medications. Additionally, we and others have found a strong association between long-term metformin treatment and a decreased risk and severity of DR in type 2 diabetic patients, regardless of glycemic control [11, 12]. Metformin probably protects against vasculopathy by reducing inflammation [13, 14]. There is, however, no concrete evidence to support the concept that metformin has an anti-inflammatory effect in the eyes of people with diabetes mellitus.

In this study, vitreous from PDR patients receiving metformin or not receiving metformin was analyzed and compared to people without diabetes. Human retinal vascular endothelial cells (hRVECs) conditioned with high glucose (HG) were used *in vitro* to aid in the understanding of probable underlying mechanisms of metformin’s actions.

## Methods

### Subjects

This is a cross-sectional, case-control study of 16 consecutive patients with a history of type 2 diabetes mellitus (T2D) > 15 years complicated by PDR and seven age-matched controls without diabetes or metformin use who underwent vitrectomy for noninflammatory ocular diseases such as idiopathic macular hole, macular pucker, and aqueous misdirection. The metformin group (n = 6) included PDR patients who had taken oral metformin for at least five years prior to our study. The non-metformin group (n = 10) included those who had never used metformin. The use of insulin or any other hypoglycemic medication concurrently was not excluded. Exclusion criteria included systemic inflammatory or immunological illnesses, the use of immunoregulatory drugs, intermittent metformin usage, a history of other retinal disorders, or previous vitreoretinal surgery.

This study adhered to the guidelines of the Declaration of Helsinki and was approved by the Institutional Review Board of Henry Ford Health System. Informed consent was obtained from each patient before the collection of the vitreous specimens. During pas plane vitrectomy, about 1–1.5 ml clear, undiluted vitreous were extracted through vitreous cutter from the central vitreous cavity before intraocular infusion. The vitreous specimens were collected into sterile Eppendorf tubes and immediately stored at −80°C until use.

## Detection of pro-inflammatory cytokines

Following the manufacturer’s instructions, a human cytokine array kit (R&D Systems, MN) was used to perform a high-throughput and semi-quantitative examination of a panel of inflammatory cytokines in the vitreous. Briefly, 0.5 ml of vitreous samples were double diluted with blocking buffer and incubated with biotinylated detection antibodies. An array membrane with pre-spotted capture antibodies was immersed in each of the vitreous specimens for incubation at 4°C overnight, then washed and developed with streptavidin-HRP conjugate. The cytokines captured on the membrane were visualized using chemiluminescent detection reagents in a ChemiDoc™ MP System (Bio-Rad, Hercules, CA). Quantitative analysis was performed using Image Lab™ (Bio-Rad, Hercules, CA).

### ELISA

The quantification of IL-8, sICAM-1, MCP-1, and VEGF in the vitreous specimens and hRVEC conditioned medium was performed with ELISA kits (R&D systems, Minneapolis, MN; or bioscience, San Diego, CA), following the manufacturer’s instructions.

### Autofluorescence of AGEs

The intensity of autofluorescence with an excitation wavelength of 360 ± 40 nm and an emission wavelength of 460 ± 40 nm was evaluated using a fluorescence spectrophotometer (SpectraMax M5; Molecular Devices, Sunnyvale, CA) to determine the amount of advanced glycation end products (AGEs) in vitreous specimens [15, 16]. Background readings from blank wells were subtracted. PierceTM BCA Protein Assay Kit was used to determine the protein concentration of each sample (Thermo Fisher Scientific, Waltham, MA). The level of autofluorescence caused by AGEs in the vitreous was expressed as fluorescence intensity per 0.1 mg protein.

### Cell culture and treatment

Primary hRVECs (ACBRI 181, Cell Systems, Kirkland, WA) were grown in EGM™-2 BulletKit medium (Lonza Inc., Allendale, NJ) at 37°C and 5% CO2, and used in the experiment before to the tenth passage. D-glucose was added at a final concentration of 30 mM to the culture medium to imitate the high glucose (HG) state found in diabetes. For normal glucose (NG) control, a medium containing 5.5 mM D-glucose was employed. Confluent hRVECs were exposed to 10 mM metformin hydrochloride (Sigma-Aldrich, St. Louis, MO) for 72 hours for treatment. The dose of 10 mM metformin was selected based on our previous study [17] as well as the preliminary results of this current study.

To assess the role of adenosine 5`monophosphate-activated protein kinase (AMPK) pathway in metformin’s effect in hRVECs, a non-specific AMPK inhibitor Compound C (Sigma-Aldrich, St. Louis, MO) was administered at a final concentration of 10 μM along with metformin. To examine how metformin affects nuclear factor kappa-light-chain-enhancer of activated B cells (NF-κB), a cocktail of NF-κB inhibitors (Sigma-Aldrich, St. Louis, MO) BMS-345541 (1 μM) and MG-132 (1 ng/mL) was added to hRVEC culture for 4 hours. Additionally, an NF-κB stimulant known as PMA (phorbol 12-myristate 13-acetate, Sigma-Aldrich, St. Louis, MO) was introduced to hRVEC culture at a concentration of 50 ng/mL for 2 hours following metformin treatment.

### Immunoblot

The cells were collected in RIPA buffer (Sigma-Aldrich, St. Louis, MO) for centrifugation. The supernatant of cell lysate was used for 6–18% SDS–PAGE. Following protein transfer, PVDF membranes were blocked in 0.2% I-Block™ (*Thermo Fisher Scientific*, Waltham, MA) and incubated overnight at 4°C with the following primary antibodies: anti- pAMPKα1 (Cell Signaling Technology, Beverly, MA), anti-NF-κB p65 (Cell Signaling Technology, Beverly, MA), anti-ICAM-1 (Santa Cruz Biotechnology, Dallas, TX. USA), and anti-β-actin (Santa Cruz Biotechnology, Dallas, TX). HRP-conjugated secondary antibodies (Jackson ImmunoResearch, West Grove, PA) were applied subsequently. The blots were then viewed by an enhanced chemiluminescence solution (*Thermo Fisher Scientific*, Waltham, MA) in ChemiDoc™ MP System (Bio-Rad, Hercules, CA). Quantification analysis was performed with Image Lab™.

### Statistical analysis

Statistical significance was assumed at *p* ≤ 0.05. Data from the cytokine array were transformed by adding a constant of 0.0003 to natural logarithm values to normalize the data distribution. The Kruskal-Wallis test was conducted to compare the difference in cytokine levels between three groups. Furthermore, Wilcoxon-rank sum test was performed for pairwise comparisons. 95% confidence interval (CI) was calculated for the mean difference between each pair of groups. To adjust p-values due to multiple testing, false discovery rate was calculated using the Benjamini-Hochberg approach. Cytokines with FDR<0.05 are considered statistically differentially expressed. Pearson correlation analysis for assessing the linear relationship between two variables was applied to detect the correlations among the vitreous inflammatory cytokines. ANOVA was used to compare numeric variables among groups of the cell study.

## Results

### Attenuated upregulation of inflammatory cytokines in the metformin group

**Table 1** summarizes the demographic and clinical characteristics of the three groups of vitreous samples. The non-metformin (19 ± 2.7 years) and metformin group (18.7 ± 2.4 years) had comparable diabetes durations and DR treatments. The 5-year median HbA1c level was considerably higher in the metformin group (10.5 ± 0.8%) than in the non-metformin group (7.5 ± 0.8%) (*p < 0*.*05*), which may indicate metformin’s relative inadequacy for glycaemic management. However, prior research by our group and others has demonstrated that metformin’s potential anti-inflammatory action is not dependent on glucose management [11, 12].

**Table 1:** Demographic features and clinical characteristics of the patients.

Characteristics	Control (n = 7)	Non-met PDR (n = 10)	Met PDR (n = 6)
Age (years)	67.9 ± 12.2	60.7 ± 3.8	54.5 ± 11.3
Sex (M: F)	3: 4	8: 2	2: 4
Race, (Black: White: Other)	0: 6: 1	5: 3: 2	4: 1: 1
Duration of diabetes (years)	--	19 ± 2.7	18.7 ± 2.4
HbA1c (%, median of 5-year)	--	7.5 ± 0.8	10.5 ± 0.8 *
Other treatment for DM, (Yes: No)			
Insulin		4: 6	5: 1
Other oral hypoglycemic agent		2: 8	1: 5
Other treatment for DR, (Yes: No)			
Pan-retinal photocoagulation		6: 4	6: 0
Focal/grid laser photocoagulation		1: 9	1: 5
Intravitreal anti-VEGF reagent		4: 6	3: 3
Intravitreal steroid		0: 10	1: 5

Control = non-diabetic group

Non-met PDR = non-metformin treated PDR group

Met PDR = metformin treated PDR group

*, Non-met PDR versus Met PDR comparison *p ≤ 0*.*05*.

**Fig 1:**
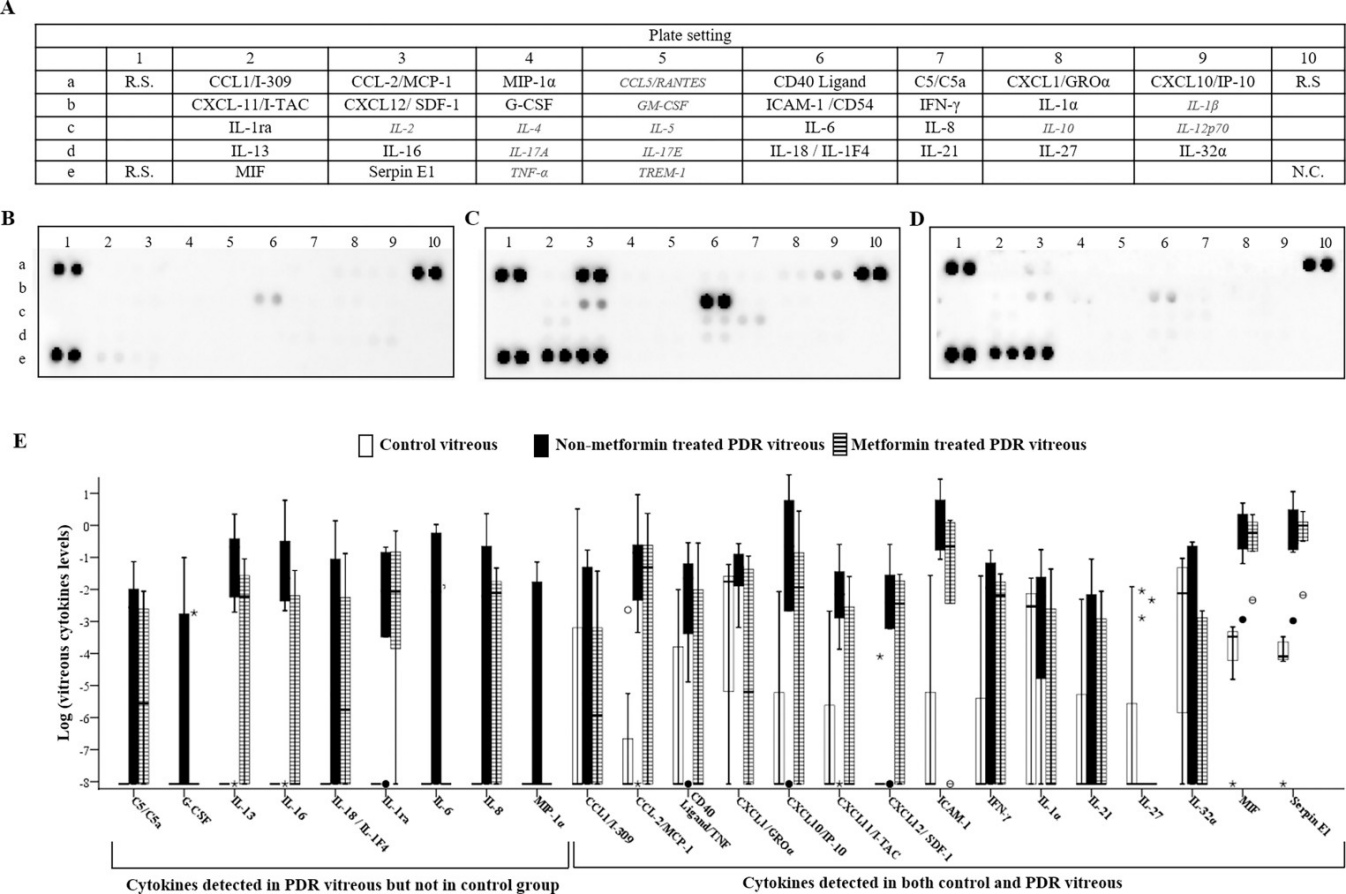
The human cytokine array analysis of multiple inflammatory cytokines and chemokines in non-diabetes control and PDR vitreous. **A,** Template demonstrating the location of various cytokine capture antibodies, reference spots (R.S.), and negative control (N.C.) of the Array. *Italic*: *12 cytokines that below the threshold of detection*. **B-D**, Representative images of the cytokine expressions in the vitreous from non-diabetic control (**B**), non-metformin treated (**C**), and metformin treated (**D**) PDR patients. **E**, The distribution of 24 measurable cytokines in the three groups.

Twenty-four (67%) of the 36 cytokines (**Fig 1A**) evaluated with the cytokine array kit were discovered in practically all specimens (**Fig 1A–1E**), while the remaining 12 (33.3%) were below the detection threshold in the majority of specimens (**Fig 1A, italic**). Nine of the twenty-four identified cytokines (9/24, 37.5%) were abundant in the PDR vitreous but not in the control (**Fig 1E; S1 Table**). The remaining 15 (62.5%) were elevated in both PDR groups compared with the control, irrespective of metformin therapy.

When the non-metformin PDR group was compared to the control group,14 out of the 24 cytokines (58%) were significantly upregulated, including C5/C5a, IL-1ra, IL-6, IL-8, IL-13, IL-16, MCP-1, CD40 Ligand/TNFSF5, CXCL10/IP-10, CXCL11/I-TAC, CXCL12/ SDF-1, ICAM-1, MIF, and Serpin E1 (all *p < 0*.*05;* **Fig 1E; S1 Table**). The comparison between the metformin group with the control revealed 3 cytokines (13%) were prominently upregulated including IL-1α, MIF, and Serpin E1 (all *p < 0*.*05;* **Fig 1E; S1 Table**). The difference was significant by logistic regression analysis (*p = 0*.*001*; **Table 2**).

**Table 2:** Logistic regression analysis of the numbers of cytokines that changed in the vitreous from PDR versus control patients for variables associated with use of metformin.

# of cytokines Vitreous groups	Significantly increased versus Control No. (%)	Non-Significantly changed versus Control No. (%)	*p value*
Non-metformin treated group	14 (58%)	10 (42%)	**0.001**
Metformin treated group	3 (13%)	21 (87%)	

**Fig 2:**
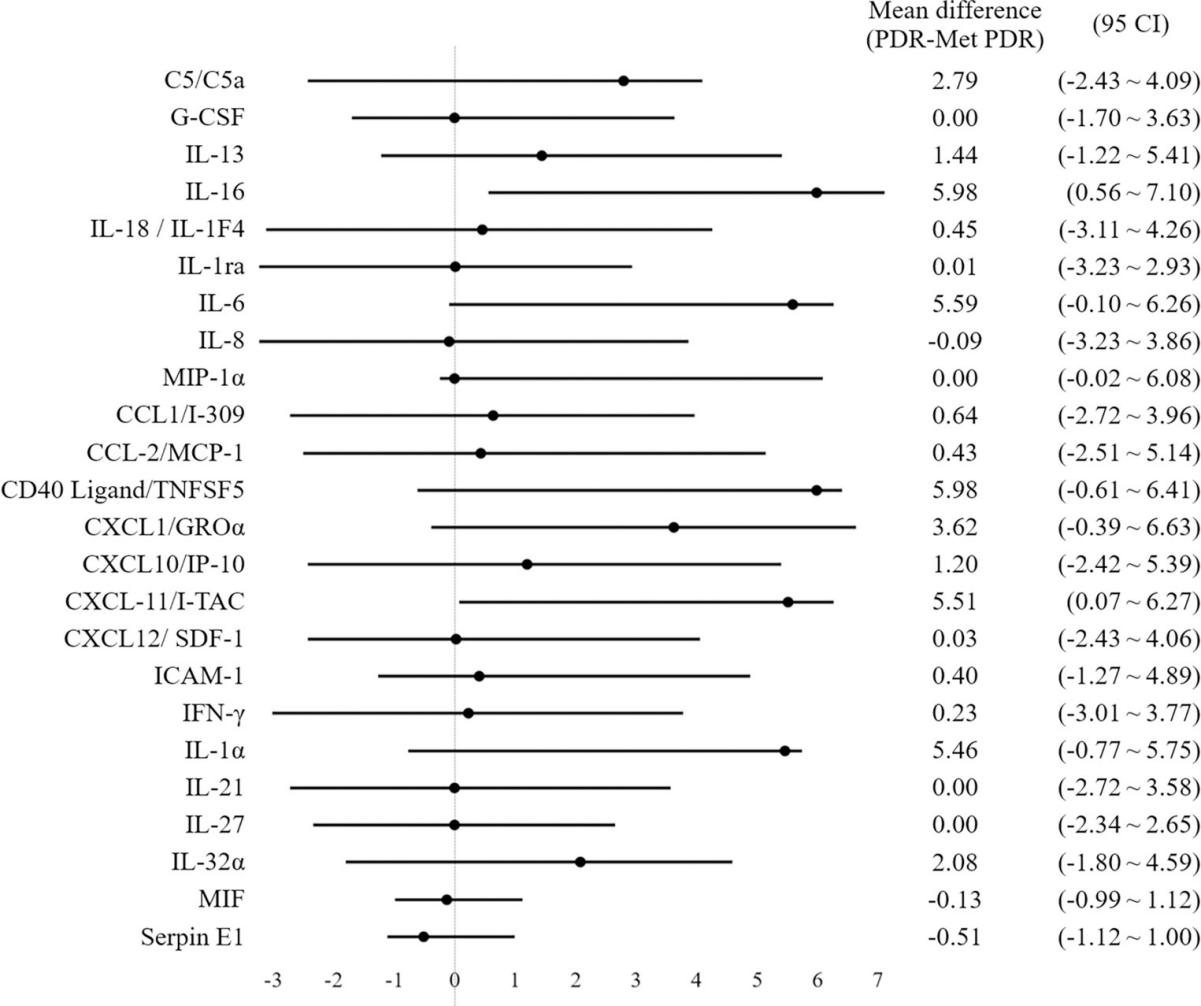
The 95% confidence interval (CI) for the mean difference between the non-metformin group and the metformin group.

The vast majority of the detected cytokines (22/24, 92%) had lower levels in the metformin group compared to the non-metformin group (**Fig 1C–1E and S1 Table**). The p values for the comparison of cytokine levels between the two groups were not statistically significant, due to a relatively small sample size and large variations (**S1 Table**). However, when the 95% CI for the mean difference between the non-metformin group and the metformin group was calculated to assess the size of difference in the true value, clinical significance was found in the difference of some of the cytokines (**Fig 2**). For IL-16 and CXCL11/I-TAC, the 95% CI does not include zero, suggesting a plausible significant difference in the true value. For C5/C5a, IL-6, CD40 Ligand/TNFSF5, CXCL1/GROα, and IL-1, the 95% CI included zero, but has a notable positive value of point estimate, which indicates the best approximation to the true value difference between the two groups.

### Significantly diminished positive correlations among the inflammatory cytokines involving MCP-1, ICAM-1, and IL-8 in the metformin group

Correlations among the detected inflammatory cytokines were assessed by Pearson’s coefficient. There were 62 significant positive correlations and no negative correlation in the non-metformin group, whereas there were 19 positive and two negative correlations in the metformin group (**Table 3**). This finding suggested extensive co-existence or co-upregulation among the cytokines in the non-metformin group, which was largely diminished in the metformin group.

**Table 3:** Pearson’s correlation coefficient for all possible pairwise combinations of the detectable vitreous cytokines within the non-metformin group and metformin group.

	C5/C5^a^	G-CSF	IL-13	IL-16	IL-18	IL-1r^a^	IL-6	IL-8	MIP-1α	CCL1/I309	MCP-1	CD40 Ligand	CXCL1/GROα	CXCL10	CXCL11	CXCL12	ICAM-1	IFN-γ	IL-1α	IL-21	IL-27	IL-32α	MIF	Serpin E1
	**Metformin treated PDR group**																							
C5/C5^a^	1									.98^b^			1.0^a^											
G-CSF		1																						
IL-13			1					.98^b^										1.0^a^						
IL-16	.64^d^		.95^a^	1																				
IL-18	.82^c^	.67^d^			1																			
IL-1r^a^					.67^d^	1								.85^d^										
IL-6							1																-.92^d^	-.95^c^
IL-8	.72^d^	.67^d^	.66^d^	.71^d^	.85^c^		.65^d^	1										.99^a^						
MIP-1α		.83^c^							1															
CCL1/I309		.73^d^			.68^d^			.88^b^		1		.83^d^	.98^b^						.83^d^	.83^d^		.82^d^		
MCP-1	.67^d^							.72^d^		.63^d^	1													
C^d^40 Ligan^d^			.77^c^	.74^d^			.72^d^					1							1.0^a^	1.0^a^		.99^a^		
CXCL1/GROα		.66^d^			.70^d^			.71^d^		.76^d^			1											
CXCL10										.75^d^				1										
CXCL11						.71^d^				.88^b^					1									
CXCL12													.87^c^		.70^d^	1							.85^d^	
ICAM-1		.85^c^			.83^c^			.85^c^		.91^b^			.86^c^	.71^d^	.69^d^	.66^d^	1							
IFN-γ												.85^c^						1						
IL-1α			.64^d^	.67^d^						.64^d^									1	1.0^a^		.99^a^		
IL-21							.75^d^													1		1.0^a^		
IL-27																				.70^d^	1			
IL-32α		.65^d^			.69^d^				.74^d^	.64^d^				.66^d^			.75^d^		.65^d^			1		
MIF	.76^d^							.71^d^			.94^a^												1	.98^b^
Serpin E1		. .71^d^						.77^c^		.77^c^	.93^a^		.64^d^			.77^c^	.77^c^						.90^b^	1
	**Non-metformin treate**^d^ **P**^d^**R group**																							

^a^ p < .0001

^b^ p < .001

^c^ p < .01

^d^ p < .05.

Particularly, MCP-1 was found to be positively correlated with C5/C5a, IL-8, CCL1/I309, MIF, and Serpin E1 in the non-metformin group (*p* values ranging from *<0*.*0001* to *<0*.*05*; **Table 3**), but not with any inflammatory cytokines in the metformin group. Similarly, in the non-metformin vitreous, ICAM-1 was significantly positively correlated with G-CSF, IL-18, IL-8, CCL1/I309, CXCL1/GROα, CXCL10, CXCL11, CXCL12, IL-32, and Serpin E1 (*p* values ranging from *<0*.*001* to *<0*.*05*; **Table 3**), but had no positive correlations in the metformin treated group. Il-8 was positively correlated with C5/C5a, G-CSF, IL-13, 1L-16, IL-18, IL-6, CCL1/I309, MCP-1, CXCL1/GROα, ICAM-1, MIF, and Serpin E1 in the non-metformin group (*p* values ranging from *<0*.*001* to *<0*.*05*; **Table 3**), but only with IL-13 and IFN-γ in the metformin group (*p <0*.*001* and *<0*.*001*, respectively; **Table 3**).

### Substantially reduced concentrations of sICAM-1, MCP-1, IL-8, and VEGF in the metformin group confirmed by ELISA

Despite its high throughput, the immunoblotting-based protein array was semi-quantitative, reporting only the relative abundance of cytokines. The exact concentrations of MCP-1, sICAM-1, and IL-8 in the same bench of vitreous specimens were quantified using ELISA. VEGF was also quantified using an ELISA as it plays a critical function in DR. The non-metformin group had significantly higher concentrations of sICAM-1 (8.4-fold), MCP (4.4-fold), IL-8 (4.1-fold), and VEGF (43.9-fold) when compared to the control group (all *p<0*.*05*, **Table 4**).

**Table 4:** Vitreous concentrations of soluble ICAM-1, MCP-1, IL-8, and VEGF in the three groups.

Cytokines	Concentrations (Mean ± S.E.)		
	Control	Non-metformin treated group	Metformin treated group
sICAM-1 (ng/mL)	7.15 ± 1.82	60.34 ± 7.97*	38.39 ± 7.14*^#^
MCP-1 (pg/mL)	1426 ± 312.33	6294.78 ± 1094.39*	3730.78 ± 659.84*^#^
IL-8 (pg/mL)	60.55 ± 8.8	247.83 ± 51.69*	95.1 ± 11.5
VEGF (pg/mL)	49 ± 39.64	2150.33 ± 942.39*	774 ± 361.91

* Non-metformin treated group or Metformin treated group versus control comparison *p* ***≤*** *0*.*05*.

^#^ Metformin treated group versus Non-metformin treated group comparison *p* ***≤*** *0*.*05*.

The metformin group demonstrated a similar tendency of increased levels of these cytokines than the control group, but to a significantly smaller extent (5.4-fold for sICAM-1, 2.6-fold for MCP-1, 1.6-fold for IL-8, and 15.8-fold for VEGF; **Table 4**). When compared with the non-metformin group, the vitreous concentrations of sICAM-1 and MCP-1 were substantially lower in the metformin group (both *p* ***≤*** *0*.*05*; **Table 4**), while those of IL-8 and VEGF were likewise lower but not statistically significant (**Table 4**). The quantities of vitreous cytokines found in our investigation were comparable to those previously reported [18–20]. These data corroborated metformin’s reduction of cytokines, emphasizing its considerable effect on ICAM-1 and MCP-1.

### Metformin treatment was associated with lower AGEs level in vitreous

**Fig 3:**
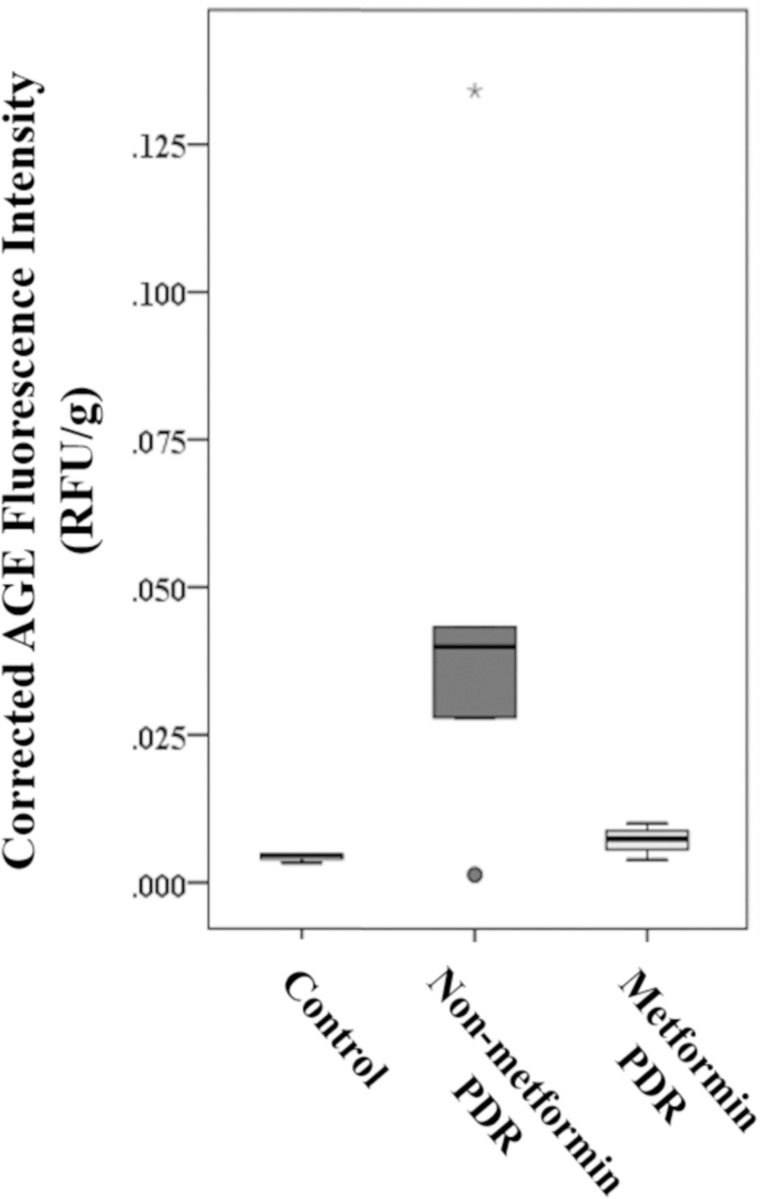
Box plot illustrating the intensity of AGE fluorescence in vitreous specimens. The non-metformin group had a more than 10-fold AGEs-associated fluorescence intensity of that in the control group, and around 6-fold of that in the metformin group. The p values for pairwise comparisons between the three groups, however, were not statistically significant.

AGEs-associated fluorescence intensity in the non-metformin group was increased by more than 10-fold compared to the control group, and around 6-fold to the metformin group, though again without statistically significance again due to large variations (**Fig 3**). The mean fluorescence levels of AGEs in the metformin group were approximately 1.6-fold of those in the control group, which was not statistically significant (**Fig 3**).

### Metformin suppresses inflammatory cytokines in hRVECs via AMPK activation and NF-B inhibition

**Fig 4:**
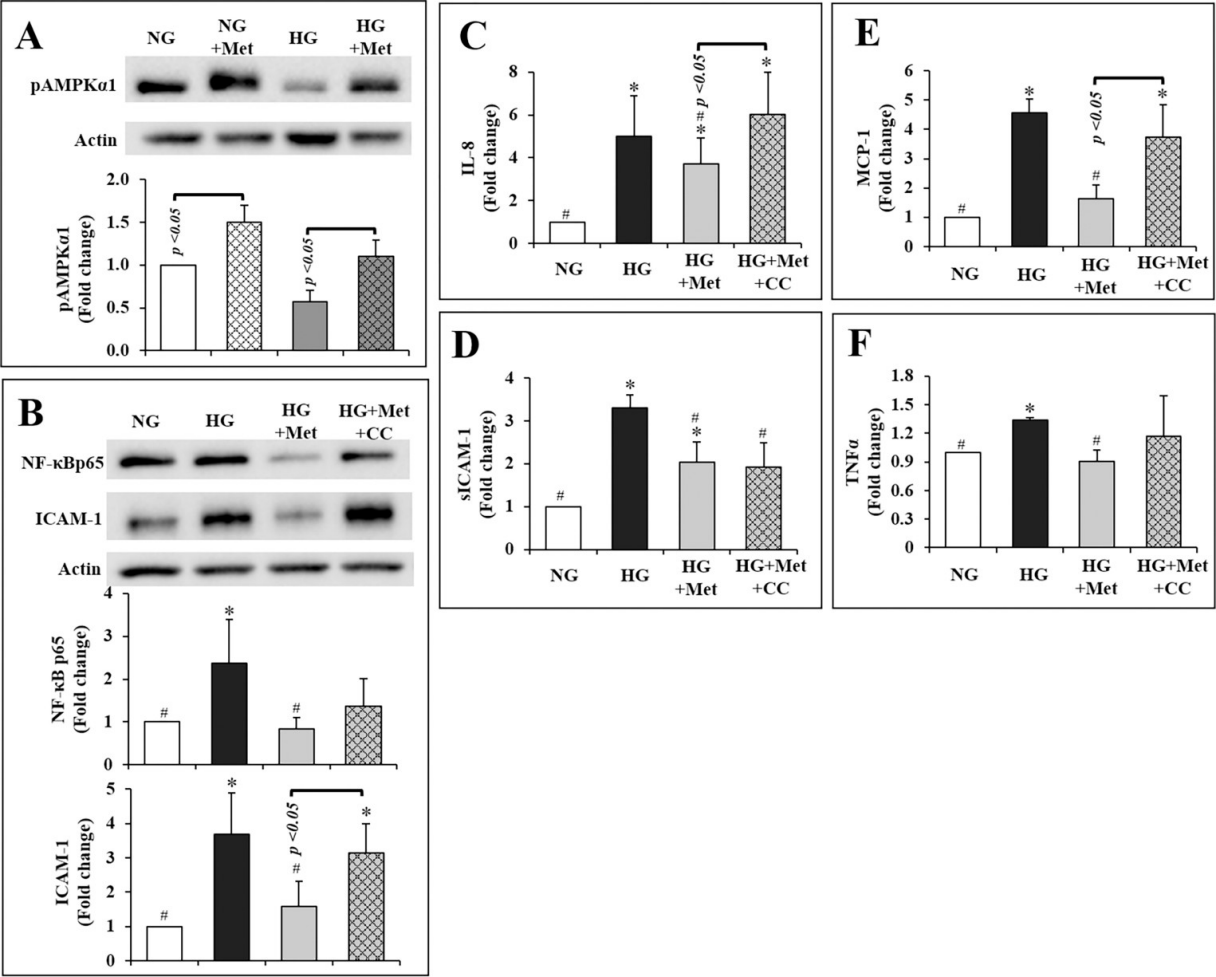
Metformin promoted pAMPKα1 in both NG and HG conditioned hRVECs (A), and inhibited HG stimulated inflammatory mediators in hRVECs through an AMPK-dependent [ICAM-1 (B), IL-8 (C), and MCP-1(E)] as well as an AMPK-independent [NF-κBp65 (B), sICAM-1 (D), and TNFα (F)] manner. The blots in (**A**) and (**B**) were representative of 3 independent experiments. All the data were expressed as mean ± SEM (n = 3) and as fold change versus NG control group. **NG**: normal glucose (5mM); **NG + Met**: normal glucose condition plus exposure to 10mM metformin for 72hr; **HG**: high glucose (30mM); **HG + Met**: high glucose condition plus exposure to 10mM metformin for 72hr.

AMPK has been shown to be critically involved in retinal inflammation [17, 21]. The active form of AMPK, pAMPKα1 was reduced by 43% in HG-conditioned hRVECs compared to NG-conditioned hRVECs (*p =* 0.08; **Fig 4A**). Metformin significantly increased pAMPK1 by 50% in NG and 91% in HG circumstances, respectively (both p 0.05; **Fig 4A**). In other words, metformin essentially restored the level of pAMPKα1 in HG condition to the NG baseline (*p =* 0.66; **Fig 4A**).

Compound C, a specific AMPK inhibitor [22], was applied along with metformin to determine whether AMPK signalling was involved in metformin’s effects *in vitro*. HG significantly increased the expression of NF-κB p65 and ICAM-1, as well as the secretion of IL-8, sICAM-1, MCP-1, and TNFα (all *p* < 0.05, **Fig 4B–4F**). Metformin completely eliminated the increase in NF-κB and TNFα induced by HG (all *p* < 0.05, **Fig 4B–4F**). Metformin also inhibited the upregulation of ICAM-1, IL-8, sICAM-1, and MCP-1 in hRVECs by HG (all *p* < 0.05, **Fig 4B–4E**). Co-treatment with compound C restored ICAM-1, IL-8 and MCP-1 to levels considerably greater than those in the HG + Met group (all *p* < 0.05, **Fig 4B, 4C and 4E**). Compound C, on the other hand, had a little or no effect on NF-κB p65, TNFα and sICAM-1 (all *p* > 0.05, **Fig 4B, 4D and 4F**). Therefore, AMPK activation was partially responsible for metformin-induced reductions in inflammatory cytokines in hRVECs.

**Fig 5:**
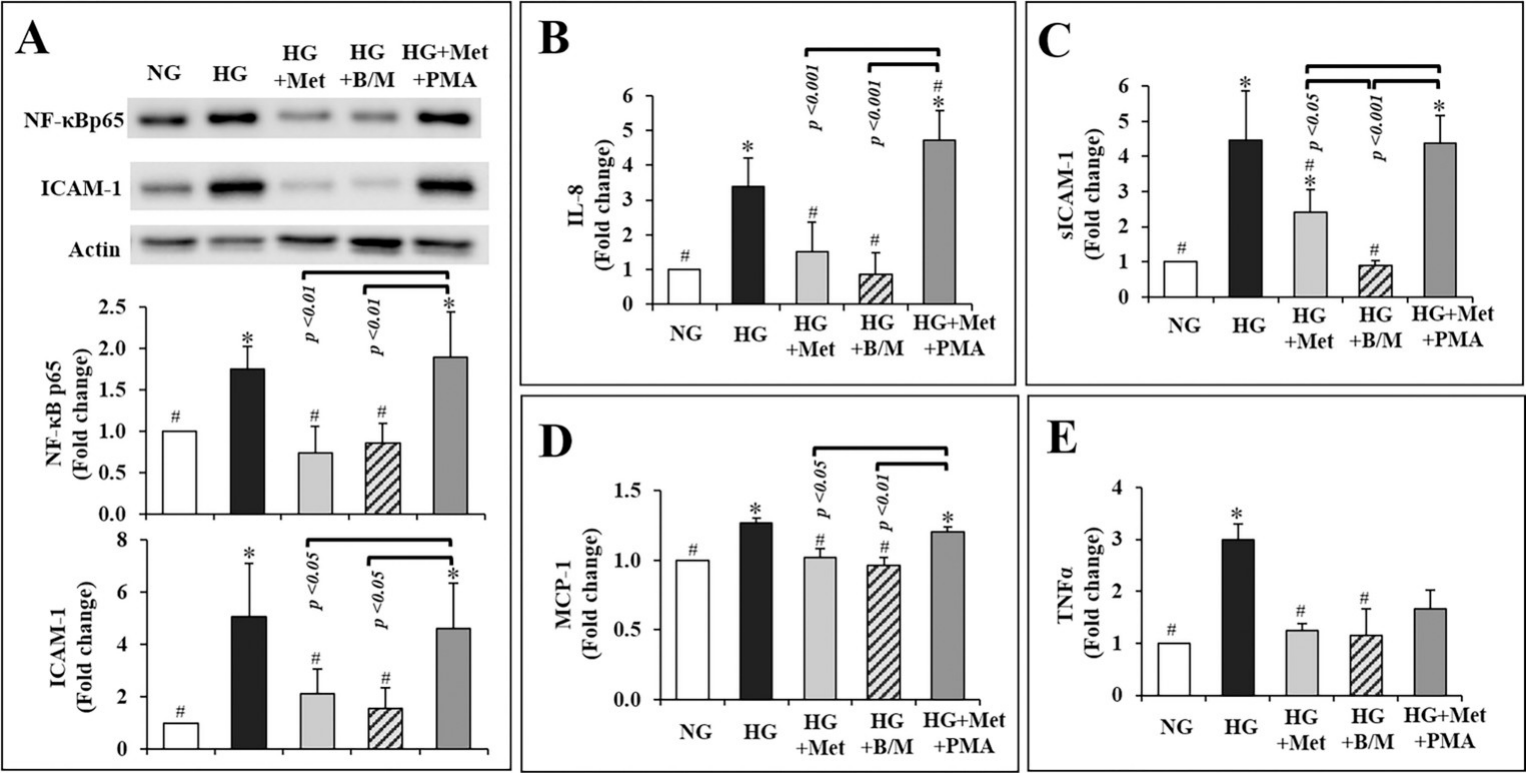
Metformin inhibited HG-induced inflammatory mediators in hRVECs with a comparable effect to that of NF-κB inhibitor cocktail (BMS-345541 and MG-132) on the expression of NF-κB p65 (A) and ICAM-1(A), and secretion of IL-8 (B), MCP-1 (D), and TNF-α (E), but not on that of sICAM-1 (C). The inhibitory effect of metformin on most of the inflammatory mediators were reversed by PMA (**A-D**), except for TNF-α (**E**). The blot in (**A**) is representative of 3 independent experiments. All the data were expressed as mean ± SEM (n = 3) and as fold change versus normal glucose control group. **NG**: normal glucose (5mM); **HG**: high glucose (30mM); **HG + Met**: high glucose condition plus exposure to 10mM metformin for 72hr; **HG + B/M**: high glucose condition plus exposure to 1 μM BMS-345541 and 1ng/mL MG-132 for 72hr; **HG + Met + PMA**: high glucose condition plus exposure to 10mM metformin for 72hr and 50 ng/mL PMA for the last 2 hr of metformin treatment.

Ingenuity pathway analysis of the vitreous cytokines modulated by metformin indicated a critical role for NF-B signalling in their modulation (Figure not shown). As shown above, metformin inhibited NF-κB p65 independent of AMPK (**Fig 4B**). A mixture of NF-κB inhibitors BMS-345541 and MG-132 was applied to compare the magnitude of effect by metformin on NF-κB and associated cytokines. The NF-κB inhibitors cocktail significantly reduced the expressions of NF-κB p65 (by 51%) and ICAM-1 (by 69%), and secretions of IL-8 (by 75%), sICAM-1 (by 80%), MCP-1 (by 24%), and TNFα (by 62%) in HG conditioned hRVECs (all *p* < 0.01; **Fig 5A–5E**). These levels reached a point where they were comparable to the NG baseline. Metformin was similarly effective in lowering the overexpression of NF-κB p65 (by 58%), ICAM-1 (by 58%), IL-8 (by 55%), MCP-1 (by 20%), and TNFα (by 58%) induced by HG (all *p* < 0.05; **Fig 5B–5E**). Metformin also inhibited sICAM-1 production (by 46%) (*p* < 0.05 VS HG group; **Fig 5C**), albeit to a lesser extent than the NF-κB inhibitors (*p* < 0.05 VS HG + B/M; **Fig 5C**).

PMA, a phorbol diester that stimulates typical protein kinase C (PKC) to enhance IκB phosphorylation and degradation [23], was also applied to assist comprehend metformin’s influence on NF-κB signaling in hRVEC. It’s worth noting that PMA considerably increased the levels of the majority of cytokines that metformin repressed (all *p* < 0.05 VS HG + Met group, **Fig 5A–5D**), except for that of TNFα (*p* = 0.62 VS HG + Met group; **Fig 5E**). This may indicate that metformin regulates NF-κB via mechanisms that are downstream of or distinct from those regulated by PMA.

## Discussion

Metformin’s possible anti-inflammatory function in diabetic vasculopathy, irrespective of glucose control, has garnered considerable study interest. We recently observed that long-term metformin use was associated with a considerably decreased severity of DR in T2D patients compared to other anti-hyperglycemic therapies [11]. Metformin also lowered retinal leukostasis in animals with STZ-induced diabetes [17], and blocked TNF-induced inflammatory cytokines in hRVECs [17]. The current investigation sought to further establish metformin’s anti-inflammatory benefits in the eyes of PDR patients and to elucidate the underlying processes in HG-conditioned hRVEC *in vitro*.

### Metformin inhibits the expression of key inflammatory markers in the vitreous of patients with PDR

It has been demonstrated that the inflammatory cytokine profile in the vitreous was considerably different from that in the serum of PDR patients, due to the local intraocular inflammation [24]. Pro-inflammatory molecules such as VEGF, ICAM-1, MCP-1, IL-6, IL-8, and TNF were consistently detected at higher levels in the vitreous of diabetic patients compared to those without diabetes, and in PDR or diabetic macular edema (DME) patients compared to non-DR patients [18, 25–28]. In animal models, the absence or suppression of key inflammatory mediators resulted in amelioration of DR-like retinopathy. For example, knockout or suppressing ICAM-1 considerably reduced retinal leukocyte infiltration and vascular leakage in diabetic mice [29, 30], whereas MCP-1 knockout animals exhibited significantly reduced retinal vasculopathy following diabetes induction [31]. This current study found a similar upregulation of multiple inflammatory cytokines in the vitreous from PDR subjects versus those from the control group without diabetes. Further, this study is the first to demonstrate a clear trend of attenuated upregulation of vitreous inflammatory cytokines associated with long-term metformin treatment. ICAM-1 and MCP-1in particular, had notably lower concentrations (Table 4) and weaker correlations (Table 3) in the metformin group versus the non-metformin group. Additionally, the metformin group had a comparable level of AGEs to the control group, which was obviously lower than the non-metformin group. Metformin has been proposed to inhibit the formation of AGEs by scavenging their dicarbonyl precursors, such as methylglyoxal, and increasing methylglyoxal metabolism [32]. Given that AGEs have been shown to stimulate NF-κB, ICAM-1, and TNFα in retinal tissue [33, 34], our finding is consistent with metformin having an anti-inflammatory effect in DR.

Metformin use has been associated with a decrease in the levels of certain inflammatory cytokines in the blood of diabetic individuals. De Jager et al. found significant reductions in plasma sVCAM-1 and E-selectin levels in T2D patients treated with metformin for 16 weeks [14]. Over a four-year follow-up period, significant reductions in plasma sVCAM-1, CRP, and sICAM-1 were seen [35]. Another study found that short-term metformin treatment (16 weeks) resulted in a substantial drop in plasma sICAM-1, but not in t-PA, TNFα, or CRP [36]. Plasma TNFα was dramatically lowered by metformin treatment in Tizazu’s findings [37]. Our findings corroborated and expanded prior research, thereby bolstering clinical support for the use of metformin in the treatment of diabetes-associated inflammation and vasculopathy.

### Metformin inhibits inflammatory cytokines partially through activation of AMPK and inhibition of NF-κB

Metformin’s molecular mechanism of action on inflammatory cytokines remains unknown. The results of prior studies varied according to the dose and length of metformin exposure, with clear distinctions between acute and chronic therapy. While activation of AMPK was demonstrated to contribute to metformin’s anti-inflammatory activity, other AMPK-independent mechanisms were also postulated. According to Zheng et al. [38], metformin upregulated LKB1/AMPK and suppressed NF-κB and Bax in hyperglycemia “pre-stressed” bovine retinal capillary endothelial cells and the retina of rats with diabetes.

Metformin inhibited HG-induced phosphorylation of Akt and PKC in ECs, without activating AMPK, as observed by Isoda et al. [13]. Their study also demonstrated that metformin prevented IL-1β–induced NF-κB activation in vascular smooth muscle cells (SMCs) through inhibiting the PI3K–Akt, but not AMPK, pathway. Cameron et al. [39] showed that metformin inhibited IκB degradation and subsequent NF-κB activation in hepatocytes independent of AMPK. Additionally, they demonstrated that metformin did not directly inhibit the upstream NF-κB regulator IKKβ and most other kinases in a cell-free kinase profiling study.

To gain a better understanding of how metformin influences the chronic inflammatory process in DR, we evaluated AMPK and NF-κB in hRVECs exposed to chronic HG (3 passages) with or without metformin exposure (72 hours). Metformin suppressed ICAM-1, IL-8, and MCP-1 in an AMPK-dependent manner (Fig 4). However, pharmacological inhibition of AMPK did not abolish metformin’s suppression of NF-κB p65, sICAM-1, and TNFα in our study (Fig 4). Metformin has previously been shown to inhibit inflammation and suppress NF-κB in vascular SMCs [13], hepatocytes [39], and gut tissue [40], independent of AMPK pathway. It is unknown which factors/mechanisms mediate metformin’s suppression of NF-B. Though metformin was proposed to reduce IκB degradation, there is disagreement over its role on the upstream kinases such as IKK [13, 39]. In our study, metformin had equivalent effects in reducing NF-κB p65, MCP-1, and TNFα to that of the NF-B inhibitors BMS-345541 and MG-132, but had a weaker effect on ICAM-1, IL-8, and sICAM-1 (Fig 5). The phorbol diester PMA, which promotes IκB degradation, reversed these effects. PMA activates a variety of PKC isoforms, including conventional (α, β1, β2, and γ) and novel (δ, ε, θ) isoforms [41], which have been reported an upstream regulator of IKK [42]. Therefore, metformin may have bypassed or acted downstream of PMA’s control of IκB in hRVECs. The molecular processes behind metformin’s suppression of p65 warrant further exploration, which is outside the scope of this study.

The dose of metformin used in our *in vitro* study (10 mM) is more than the plasma levels (between 10 to 40 μM) attained following a therapeutic dose in diabetes humans or animals [43]. The long incubation time of 72 hours with metformin also do not appropriately match *in vivo* conditions, given a short half-life of metformin *in vivo*. Previously published investigations utilizing hRVEC [17], HUVEC [44], or intact hepatocytes [45] required a significantly greater dose of metformin (5–50 mM) to induce cellular responses other than glucose control. It is important to know that the sensitivity to metformin’s effects varies greatly depending on the exposed cell’s metabolic state and nutrient availability. In vitro culture conditions do not accurately replicate the in vivo microenvironment and contain excessive nutrients such as glucose, serine, and glutamine, all of which reduce metformin sensitivity [46, 47]. There was no evidence of cellular stress or apoptosis in our trials with this dose of metformin in hRVECs [17].

### Strength and limitation of our study

This study established for the first time that metformin has a potential anti-inflammatory effect in diabetic retinopathy. We acknowledge the inherent restriction of doing a cross-sectional clinical study with a small sample size due to the scarcity of human tissue. This proof-of-concept study revealed not a direct cause-and-effect relationship between metformin and reduced inflammation in the vitreous of PDR patients, but rather a substantial association between metformin and attenuated local inflammation. Due to the small sample size, this study was planned and powered to identify a trend of change rather than a statistically significant difference. We used three distinct sensitivity analyses to attempt to minimize these constraints as much as practicable. First, a logistic regression analysis revealed significantly fewer cytokines that were up-regulated in the metformin group compared to the non-metformin group; second, Pearson’s coefficient revealed significantly diminished co-relations involving critical cytokines in the metformin group; and third, a highly sensitive ELISA assay demonstrated significant decrease in sICAM-1 and MCP-1 in the metformin group. Our in vitro investigations demonstrated that metformin inhibits NF-κB in an AMPK-independent manner and that AMPK and NF-κB cooperate to regulate ICAM-1, MCP-1, IL-8, sICAM-1, and TNFα. Because the primary objective of this investigation was to establish metformin’s potential anti-inflammatory function in the eyes, the signaling cascade regulating metformin’s modulation of AMPK and NF-κB p65 was not examined further in this work. By demonstrating that metformin inhibited multiple inflammatory cytokines in the vitreous of PDR patients and human RVECs, this study established a significant anti-inflammatory role for metformin in human eyes, which helped to explain the previously reported association between long-term metformin use and decreased severity of DR in T2D patients [11].

## Conclusion

This study established for the first time that metformin has an anti-inflammatory effect in the vitreous of PDR patients. Long-term metformin treatment was associated with substantially attenuated cytokine upregulation and remarkable elimination of co-relations between these cytokines. Metformin’s anti-inflammatory effect is separate from its glycaemic control effect. Metformin substantially reversed the upregulation of ICAM-1, sICAM-1, MCP-1, IL-8, and TNFα in high glucose-conditioned hRVECs, in part through activating AMPK. Metformin decreased NF-κB activity independent of AMPK, albeit the signaling cascade underlying this inhibition remains unknown. Metformin’s anti-inflammatory activity in the eyes of DR patients is consistent with clinical observations that metformin therapy was related with decreased severity of DR independent of A1C level. Our findings motivated us to conduct additional research on metformin for inflammatory suppression in the very early stages of DR, both alone and in conjunction with other glycemic control drugs.

## Supporting information

S1 Table Group description and comparison of the twenty-four cytokines detected with the cytokine array in the three groups of vitreous samples. (DOCX)

S1 Raw images (PDF)
