# Supplementary material for: Metformin suppresses pro-inflammatory cytokines in vitreous of diabetes patients and human retinal vascular endothelium
Source: PLoS One. 2022 Jul 8;17(7):e0268451. doi: 10.1371/journal.pone.0268451 (PMC9269956; doi:10.1371/journal.pone.0268451)
Supplement: S1 Table — (DOCX) [file pone.0268451.s001.docx]

**S Table 1.** Group description and comparison of the twenty-four cytokines detected with the cytokine array in the three groups of vitreous samples.

|  | **Cytokines** | **Mean**^1^ **(Std. Dev.) log** | | | **Kruskal-Wallis** | **Wilcoxon Rank Sum test**  **(FDR adjusted)** | | |
| --- | --- | --- | --- | --- | --- | --- | --- | --- |
|  |  | **Control (n=7)** | **Non-met PDR (n=10)** | **Met PDR (n=6)** | **p.FDR^2^** | **Non-met PDR vs Control** | **Met PDR vs Control** | **Non-met PDR vs Met PDR** |
| Cytokines detected in PDR vitreous but not in control group | C5/C5a | -8.11 (0.00) | -4.72 (2.95) | -5.55 (2.82) | 0.063 | **0.032** | 0.136 | 0.772 |
|  | G-CSF | -8.11 (0.00) | -6.32 (2.92) | -7.28 (2.03) | 0.343 | 0.182 | 0.510 | 0.868 |
|  | IL-13 | -8.11 (0.00) | -2.10 (2.31) | -4.20 (3.07) | 0.009 | **0.006** | 0.085 | 0.382 |
|  | IL-16 | -8.11 (0.00) | -2.33 (2.30) | -6.16 (3.02) | 0.009 | **0.006** | 0.247 | 0.382 |
|  | IL-18 | -8.11 (0.00) | -4.80 (3.55) | -5.37 (3.09) | 0.122 | 0.061 | 0.136 | 0.868 |
|  | IL-1ra | -8.11 (0.00) | -3.39 (2.63) | -3.24 (2.72) | 0.025 | **0.013** | **0.043** | 0.957 |
|  | IL-6 | -8.11 (0.00) | -4.07 (3.56) | -7.15 (2.35) | 0.043 | **0.032** | 0.510 | 0.382 |
|  | IL-8 | -8.11 (0.00) | -3.92 (3.33) | -4.23 (3.02) | 0.043 | **0.022** | 0.085 | 0.868 |
|  | MIP-1α | -8.11 (0.00) | -5.69 (3.13) | -8.11 (0.00) | 0.058 | 0.072 | 1.000 | 0.420 |
| Cytokines detected in both control and PDR vitreous | CCL1/I-309 | -5.67 (3.25) | -5.04 (3.27) | -5.66 (2.78) | 0.825 | 0.695 | 1.000 | 0.868 |
|  | MCP-1 | -7.01 (2.00) | -2.23 (2.35) | -3.55 (3.60) | 0.042 | **0.013** | 0.170 | 0.868 |
|  | CD40 Ligand | -6.17 (2.49) | -3.11 (2.16) | -6.00 (3.30) | 0.063 | **0.032** | 1.000 | 0.423 |
|  | CXCL1/GROα | -3.87 (2.92) | -1.95 (0.72) | -5.07 (3.35) | 0.119 | 0.102 | 0.820 | 0.382 |
|  | CXCL10/IP-10 | -6.55 (2.66) | -2.28 (3.31) | -3.76 (3.47) | 0.063 | **0.032** | 0.215 | 0.765 |
|  | CXCL11/I-TAC | -6.74 (2.35) | -3.08 (1.98) | -6.25 (2.90) | 0.042 | **0.022** | 0.820 | 0.382 |
|  | CXCL12/SDF-1 | -7.58 (1.40) | -3.55 (2.53) | -4.36 (2.94) | 0.043 | **0.018** | 0.136 | 0.868 |
|  | ICAM-1 | -6.48 (2.79) | -0.58 (0.82) | -2.39 (2.94) | 0.009 | **0.005** | 0.136 | 0.632 |
|  | IFN-γ | -6.54 (2.71) | -3.91 (2.98) | -4.29 (2.97) | 0.201 | 0.108 | 0.307 | 0.868 |
|  | IL-1α | -4.94 (2.98) | -3.73 (2.55) | -6.22 (2.95) | 0.292 | 0.363 | 0.656 | 0.436 |
|  | IL-21 | -6.60 (2.58) | -5.95 (2.92) | -6.38 (2.70) | 0.825 | 0.609 | 1.000 | 0.868 |
|  | IL-27 | -6.62 (2.56) | -7.07 (2.21) | -7.22 (2.18) | 0.868 | 0.777 | 0.820 | 0.957 |
|  | IL-32α | -3.99 (2.93) | -5.07 (3.32) | -6.47 (2.55) | 0.381 | 0.800 | 0.220 | 0.774 |
|  | MIF | -4.56 (1.65) | -1.02 (0.98) | -1.09 (0.90) | 0.009 | **0.001** | **0.013** | 0.868 |
|  | Serpin E1 | -4.76 (1.50) | -0.99 (1.04) | -0.93 (0.87) | 0.009 | **0.001** | **0.013** | 0.868 |

Control=non-diabetic group

Non-met PDR= non-metformin treated PDR group

Met PDR= metformin treated PDR group

^1^ Cytokine expression was transformed as log (cytokine + 0.0003), then standardized.

^2^ FDR adjusted p values from Kruskal-Wallis test.
