# Supplementary material for: Metformin suppresses pro-inflammatory cytokines in vitreous of diabetes patients and human retinal vascular endothelium
Source: PLoS One. 2022 Jul 8;17(7):e0268451. doi: 10.1371/journal.pone.0268451 (PMC9269956; doi:10.1371/journal.pone.0268451)
Supplement: S1 Raw images — (PDF) [file pone.0268451.s002.pdf]

Human Cytokine Array
non-diabetic control

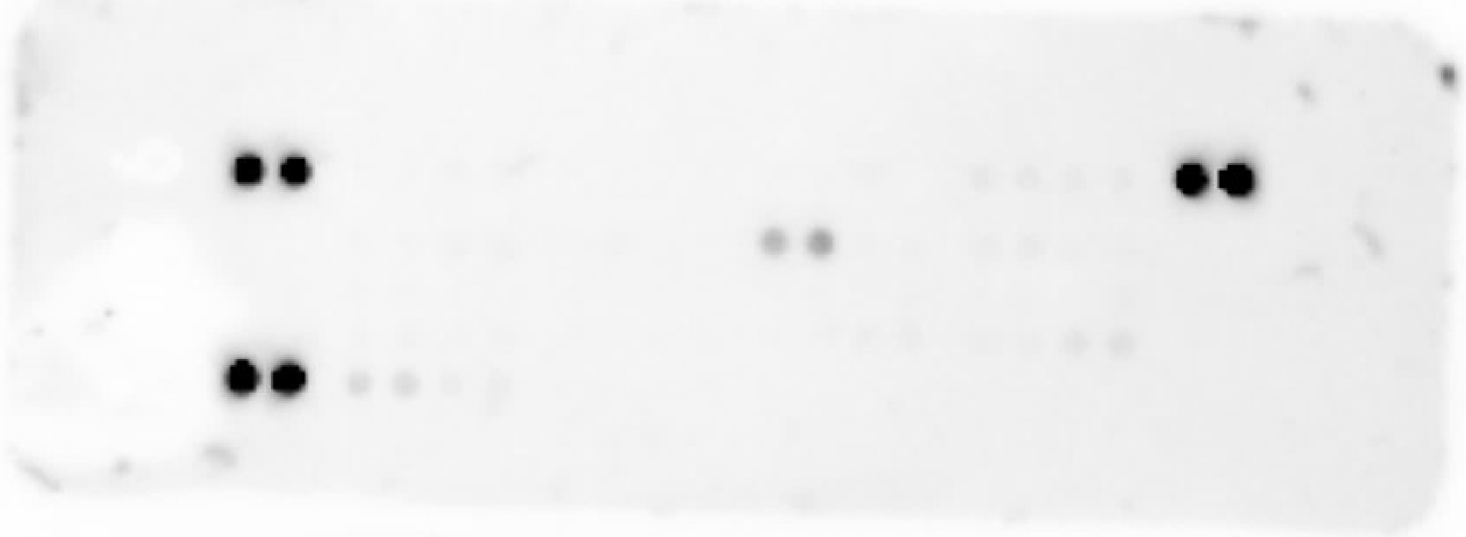

Human Cytokine Array
non-metformin treated PDR

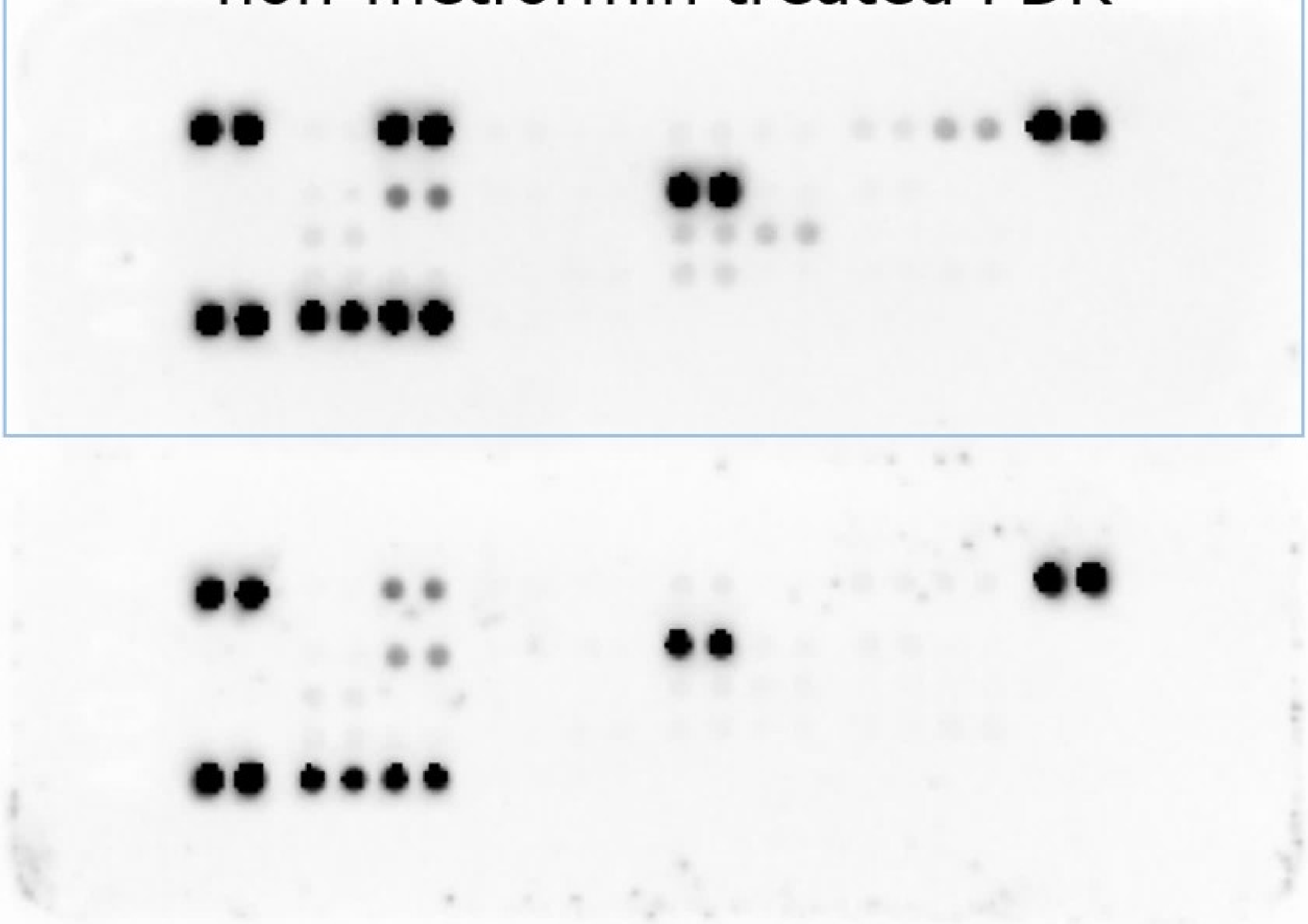

Human Cytokine Array
Metformin treated PDR

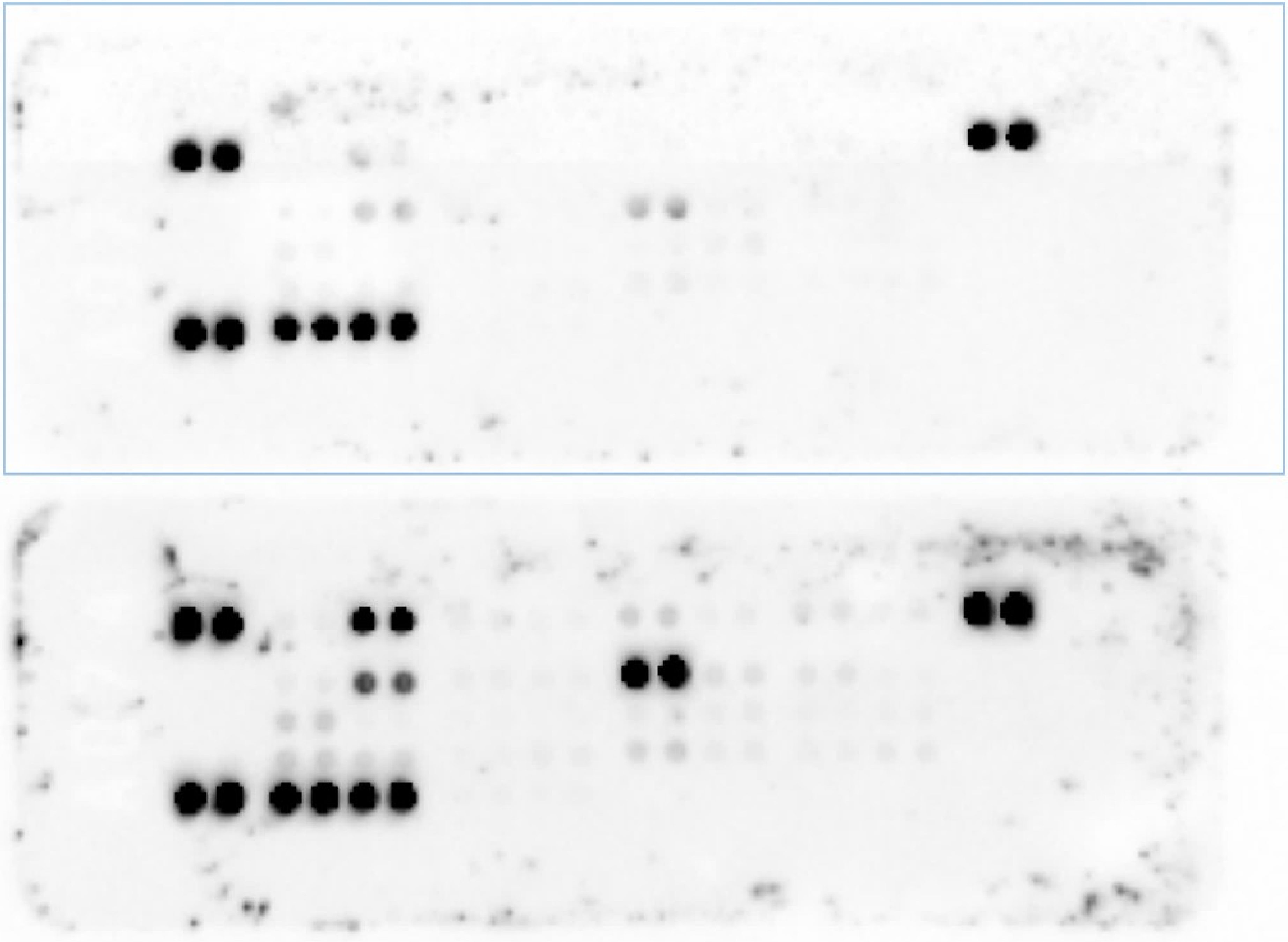

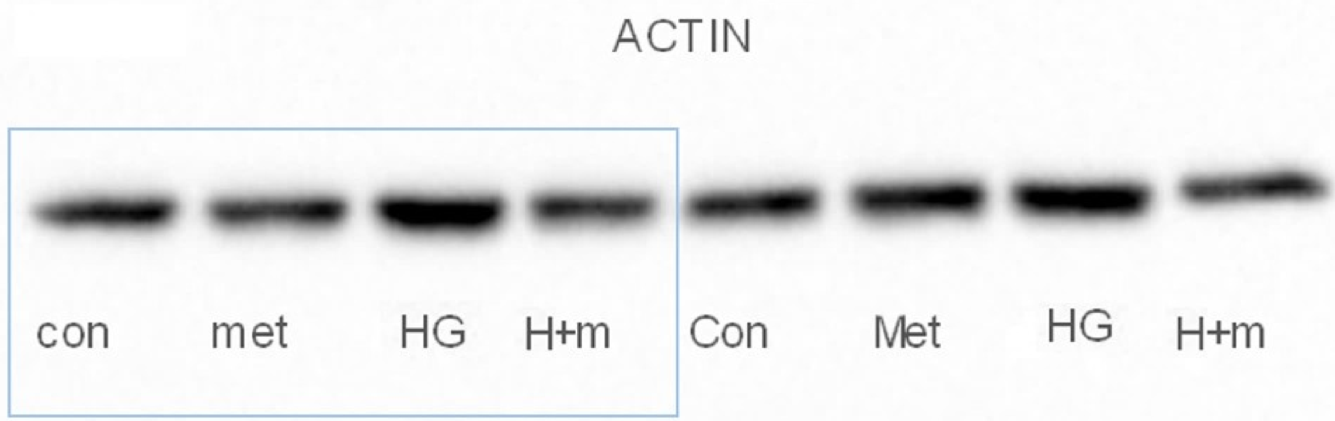
ACTIN
con
met
HG
H+m
Con
Met
HG
H+m

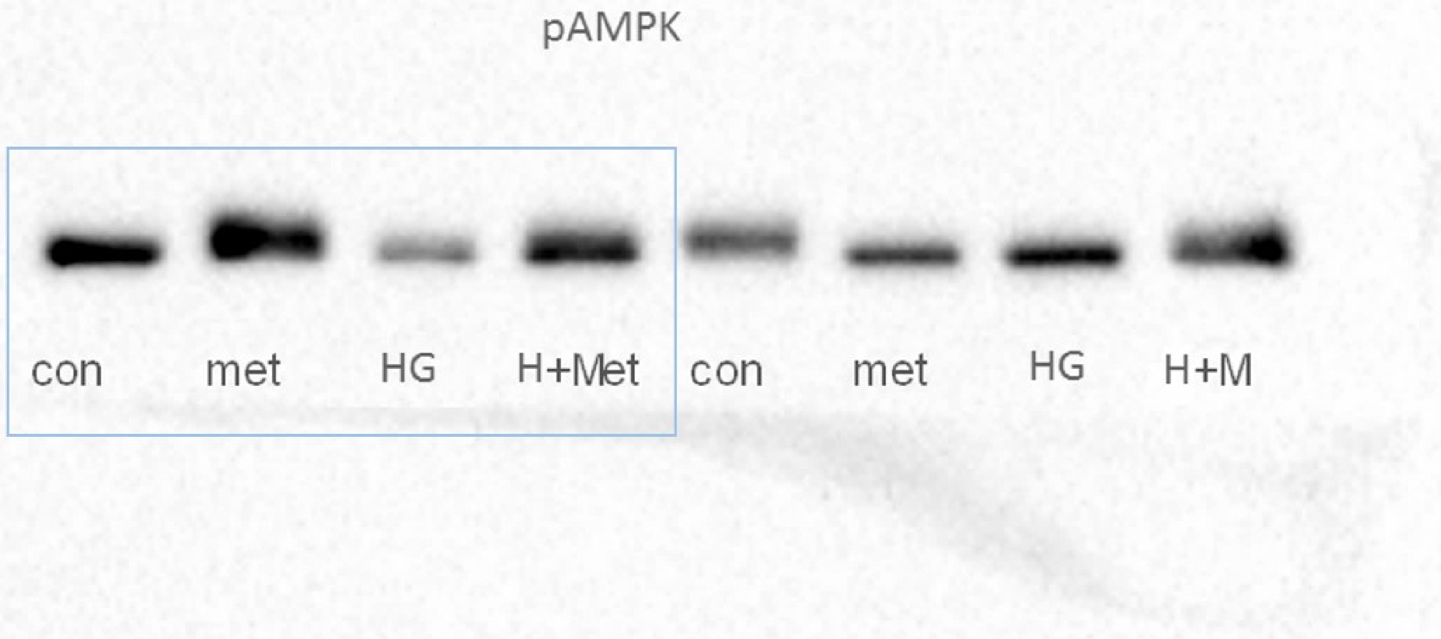
pAMPK
con
met
HG
H+Met
con
met
HG
H+M

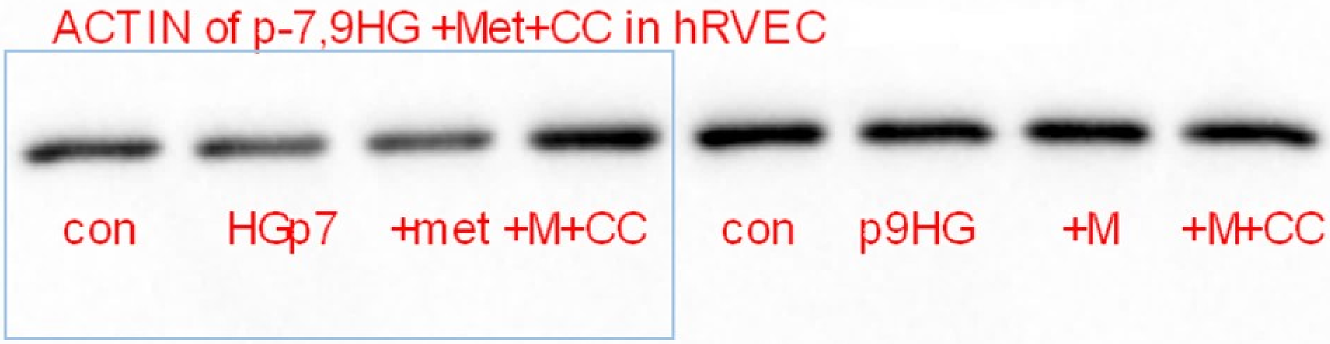
ACTIN of p-7,9HG +Met+CC in hRVEC
con
HGp7
+met
+M+CC
con
p9HG
+M
+M+CC

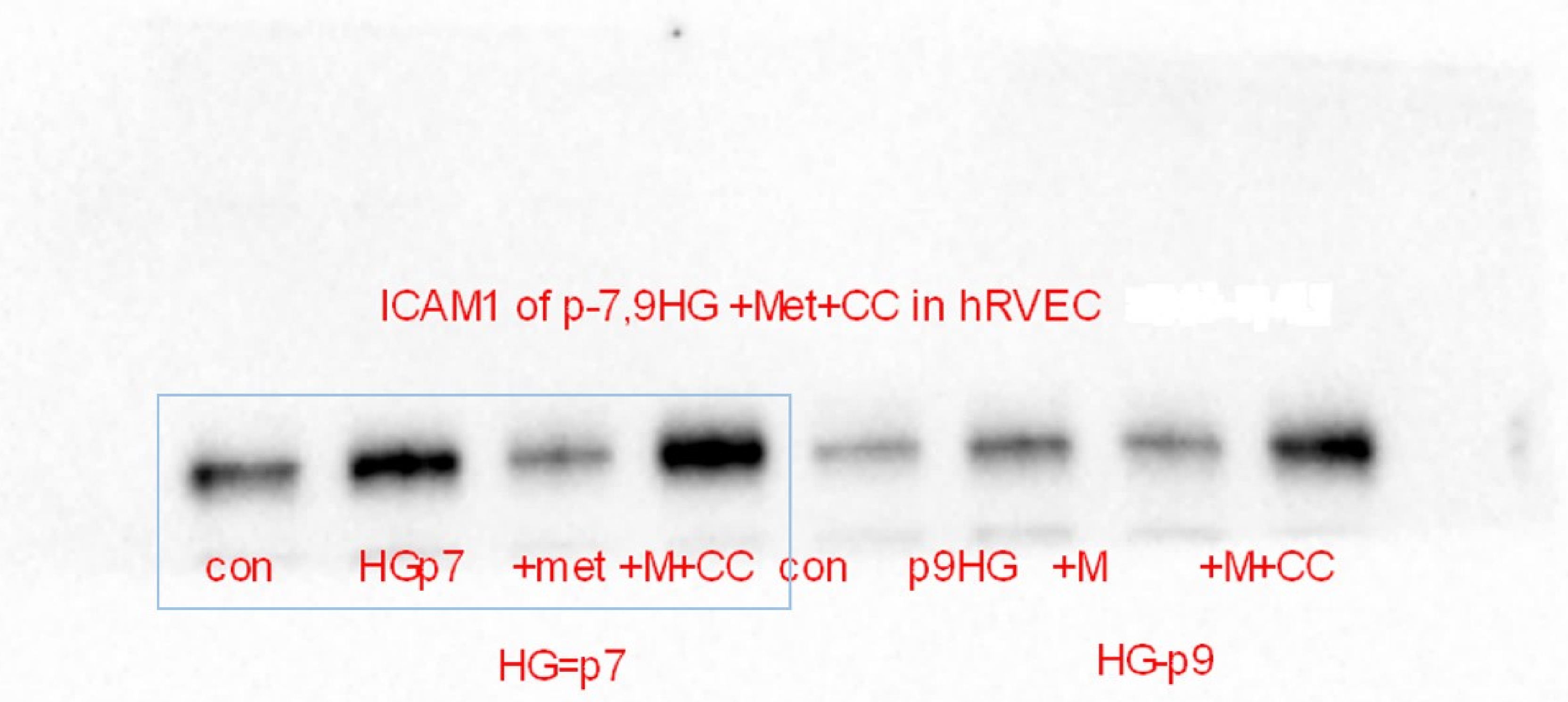
ICAM1 of p-7,9HG +Met+CC in hRVEC
con
HGp7
+met
+M+CC
con
p9HG
+M
+M+CC
HG=p7
HG-p9

p-NFkB of p-7,9HG +Met+CC in hRVEC

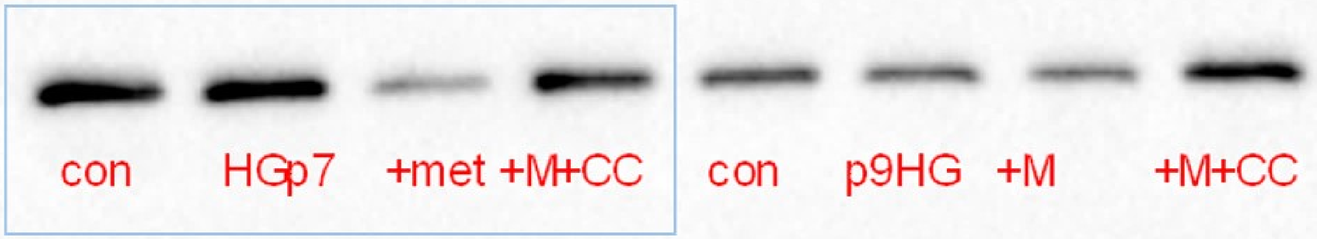

Actin of P7 HG+Met+B/M/P

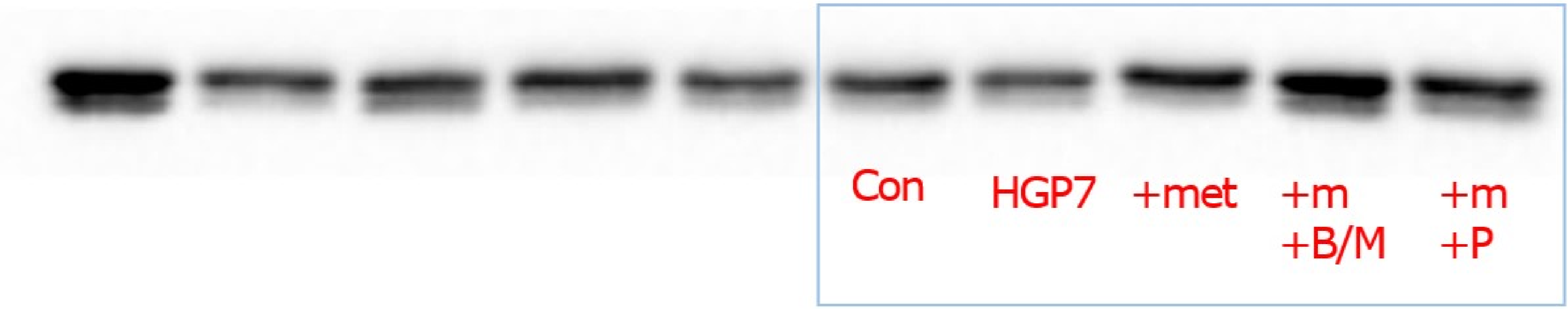

ICAM-1 , P7 HG+Met+B/M/P

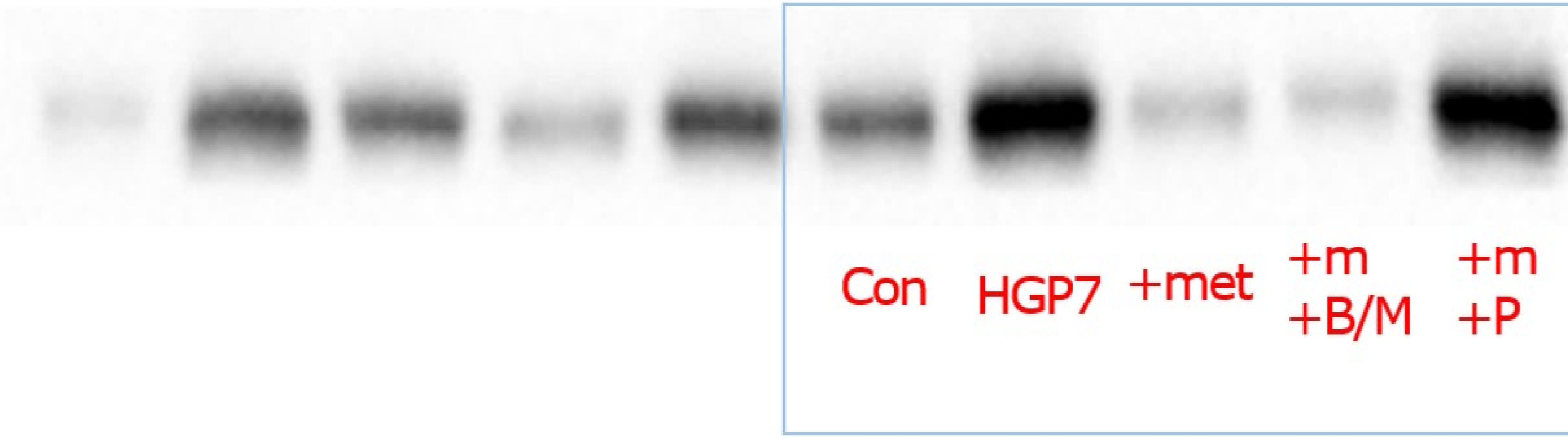

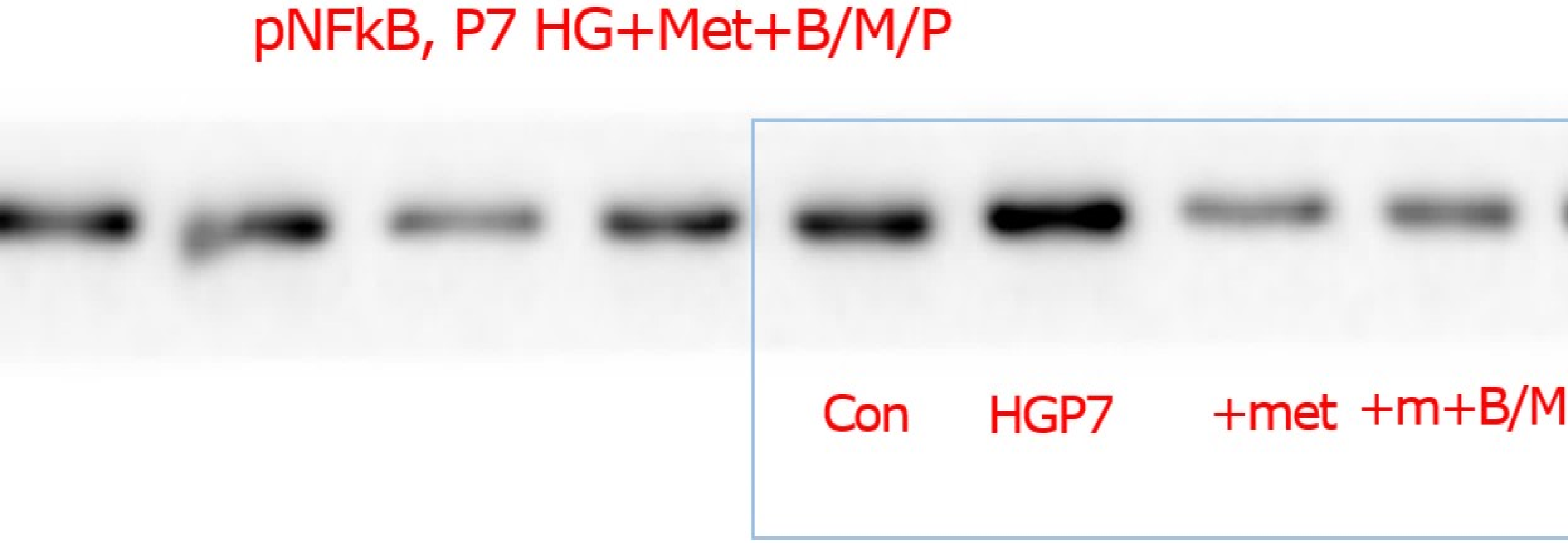
pNFkB, P7 HG+Met+B/M/P
Con
HGP7
+met
+m+B/M
